# Improving cognition and perception towards failure: a conceptual replication study

**DOI:** 10.3389/fpsyg.2025.1650136

**Published:** 2025-12-08

**Authors:** Tanmay Sinha

**Affiliations:** National Institute of Education, Nanyang Technological University, Singapore, Singapore

**Keywords:** failure, goal orientation, growth mindset, learning design, utility value

## Abstract

Despite its pedagogical value, failure is not often desired by students. To address this motivational barrier, I report a conceptual replication study that explored the synergistic effects of combining design principles from two distinct research traditions—growth mindset and utility value—to improve students’ dispositions toward failure. Using a single-group pre-post design, *N* = 68 lower secondary students from Singapore engaged in a pilot intervention involving prediction-explanation cycles on growth mindset myths along with evaluation of peer quotations reframing failure. Mixed methods analyses showed that this brief intervention was successful in significantly improving students’ learning goal orientation and attitude towards mistakes (strong effect sizes), representing rapid change in traditionally difficult-to-influence areas in education. Conversely, deeper cognitive orientations pertaining to beliefs about ability and the utility of failure showed non-significant improvements (weak to moderate effects). These results call on educators to proactively design repeated sense making opportunities involving reflections and vicarious learning to improve students’ cognition and perception regarding failure.

## 1 Introduction

Although empirical evidence affirms that deliberately designed instances of failure are critical catalysts for deep learning (e.g., Sinha and Kapur, 2021; Wong and Lim, 2022), these failure-prone situations are frequently avoided by students and remain highly unpreferred (Pan et al., 2020). This avoidance is rooted in the psychological and social costs associated with failure: students typically interpret the required greater mental effort as unduly taxing (David et al., 2024) and perceive failure as a threat to self-worth or an indicator of fixed ability, often leading to poorer learning outcomes (Deslauriers et al., 2019). This creates a crucial disconnect where students prioritize task avoidance over the challenging risk-taking necessary for mastery. To address this persistent motivation barrier, the present work therefore asks—how can we make failure more desired and help students develop the dispositions to view failure more positively? In the current conceptual replication, I utilize two strong motivational interventions (*growth mindset*, *utility value*) within an integrated pedagogical design offering opportunities for prediction, reflection and vicarious learning through peer examples, in the service of enhancing students’ *learning goal orientation* (RQ1), *attitude towards mistakes* (RQ2), *beliefs about mental ability* (RQ3) and *usefulness of failure* (RQ4).

While *growth mindset* (Dweck, 2006; Yeager et al., 2019) and *utility value* (Eccles-Parsons et al., 1983; Hulleman and Harackiewicz, 2021) interventions have often been studied independently, I argue that their underlying mechanisms are conceptually synergistic. A *growth mindset* provides the cognitive framework for interpreting failure as opportunities for improvement, whereas *utility value* influences the motivational rationale for engaging with those challenging opportunities. When combined, they can be posited to create a feedback loop—growth-oriented beliefs reduce the threat of failure, allowing students to perceive its instrumental value, while recognizing failure’s utility reinforces beliefs in the potential for growth (Zeeb and Voss, 2025). This integrated hypothesized mechanism underpins the proposed learning design, which seeks to make failure both intellectually meaningful and personally worthwhile for lower secondary students in Singaporean schooling contexts.

## 2 Theoretical background

### 2.1 Growth mindset

My initial strategy to promote a positive perception of failure focuses on cultivating a growth mindset, a well-established psychological concept that has attracted significant interest in recent years (Dweck, 2006). When students believe that their abilities can be developed through effort, they are more likely to adopt a *learning goal orientation* (Button et al., 1996; Yeager et al., 2019). This shift encourages them to view challenging tasks as opportunities for growth rather than sources of anxiety. As a result, students are motivated to engage with difficult material, which may enhance their willingness to persist in the face of setbacks (Burnette et al., 2023). Additionally, a growth mindset is also expected to influence students’ *attitudes toward mistakes* (Leighton et al., 2015)—they begin to see failures as integral to the learning process, rather than as indicators of their worth or abilities. This perspective helps reduce the fear of failure and embarrassment, promoting a more resilient approach to challenges (Yeager and Dweck, 2012).

At the intersection of mindset and achievement motivation theory (Wigfield et al., 2021), expectancies—defined as beliefs about one’s future capabilities and likelihood of success in an upcoming task—play a critical role in driving goal-directed behaviors. While originating from distinct research traditions, these two frameworks are conceptually linked by how students’ confidence in their ability to learn impacts their *beliefs about mental ability* and the *utility of failure* (Zeeb and Voss, 2025). A student operating under a fixed mindset (the belief that ability is innate and static) will tend to hold a low or rigid expectancy for future success after encountering failure, as they interpret the mistake as proof of a fundamental, unchangeable limitation. However, growth mindset interventions hold strong potential to directly manipulate this antecedent belief. When students internalize the idea that their skills can improve, they are more likely to perceive failure as sources of insight that inform their learning strategies (Sinha et al., 2021). This recognition can lead to a greater appreciation for the iterative nature of learning, where mistakes are seen as steps toward mastery (Dweck, 2006). Such a shift fundamentally alters the expectancy calculation too: the student is more likely to hold a higher, effort-contingent expectancy for success. This enhanced expectancy, coupled with the new framework for interpreting struggle, I posit will lead to greater persistence, a healthier attitude towards mistakes, and improved utility of failure. These principles collectively inform the design of innovative teaching strategies in the present work to provoke sensemaking around growth mindset principles and foster a healthier relationship with failure.

Previous student-focused growth mindset interventions (e.g., see Burnette et al., 2023 for a synthesis) exhibit limitations, including the use of learning materials that are either strictly domain-general or domain-specific, and often rely on passive instruction. My approach improves upon these constraints by integrating growth mindset and utility value principles (discussed in Section 2.2), employing a student-centered pedagogy (prediction-explanation cycles and peer evaluation) to provoke active sensemaking. Furthermore, I address measurement gaps by including new pre-post dispositional measures alongside qualitative data, testing the efficacy of this enhanced model in a new cultural context beyond European or American contexts.

### 2.2 Utility value

My complementary strategy to shift students’ perceptions of failure focuses on emphasizing its utility value, a vital component of motivational interventions grounded in expectancy-value theory (Eccles-Parsons et al., 1983). Utility value (Hulleman and Harackiewicz, 2021) is formally defined as the perceived importance, usefulness, and relevance of engaging in a task for the attainment of future goals, both proximal (e.g., passing a test) and distal (e.g., career aspirations). This theory posits that students’ motivation to engage in learning tasks is often influenced by their expectations of success and the perceived usefulness of the tasks. Research indicates that utility value interventions can impact performance, as students often view learning tasks through the emotional and psychological implications of success and failure (Harackiewicz and Priniski, 2018). Specifically, in challenging learning contexts, students may develop negative perceptions that can undermine their overall valuation of the learning process, for instance concerns about the emotional toll of failure on self-worth, the substantial effort required for mastering complex problems, and the trade-offs between engaging in failure-prone tasks versus more structured, familiar learning methods (Wigfield et al., 2021).

Former utility value interventions have primarily focused on enhancing students’ beliefs regarding the relevance and applicability of academic tasks (Hulleman and Harackiewicz, 2021). However, my approach uniquely addresses the perceived value of failure itself. I operationalize this shift by reframing failure as an instrumental resource that possesses high utility for learning and future goal attainment. Through an evaluation and explanation cycle centered on peer quotations, I aim to cultivate an understanding of the advantages associated with failure, namely, its capacity to provide diagnostic feedback, highlight knowledge gaps, and signal the necessity of strategy revision or increased effort. This reframing mechanism directly connects the often negative experiences associated with failure to a positive, future-oriented outcome, and is thereby posited to improve students’ goal orientation, attitude towards mistakes, and challenge fixed beliefs about their mental abilities. Empirical evidence supports the efficacy of such interventions, demonstrating that enhancing utility value through personal relevance can lead to greater motivation, engagement levels and performance (e.g., Gaspard et al., 2021; Rosenzweig et al., 2020).

### 2.3 Prior study

Building on these two aforementioned theoretical frameworks, Sinha (in press) carried out a quasi-experimental study with *N* = 170 upper secondary students (grade 9, age 14–15) in Singapore with a similar demographic distribution in terms of gender and ethnicity as the present sample. Whole class sections were allocated to either of the two experimental conditions (*growth mindset* or *utility value*) as preparation for learning from a math task, or a *control* condition that offered no such preparation. The *growth mindset* materials focused on both domain-general and domain (math)-specific myths and utilized the pedagogical design of repeated prediction-explanation cycles. A math-specific myth could be, for instance, *“the faster you solve math problems, the better you are at mathematics”* or *“math is a man’s world.”* Students predicted whether such statements were true or false before they were presented with a brief explanation about the myth. The *utility value* materials nudged students to evaluate peer quotations of reframing failure across formal learning contexts and write their own quotations for future peers. Mixed-methods data analyses showed that exposure to the *growth mindset* and *utility value* intervention materials (i) improved students’ beliefs about failure and math expectancies relative to their baselines, as adjudged via self-reported improvements and open-ended reflections, (ii) compared to the *control* condition, resulted in similar persistence behaviors (proxied via number of generated solutions) and posttest performance in the follow-up math task, as adjudged via ANCOVAs controlling for prior mathematics knowledge.

### 2.4 The present replication context

Drawing on this baseline work, here I designed an observational study with lower secondary school students (grade 7) who have just transitioned from primary school and face new academic expectations, social dynamics and increased independence. Given that the *growth mindset* and *utility value* intervention materials produced beneficial outcomes independently in Sinha (in press), I was interested in exploring their combined (and potentially synergistic) effects. Owing to emphasis on both viewing challenges as learning opportunities as well as connecting the relevance of failure to their academic lives and future aspirations, I posit that integrating the two interventions may better scaffold students during their critical transition period.

The present intervention materials were administered 2 weeks prior to a four-day cohort camp organized by the school aimed at enhancing resilience, where students would have to engage in team-building exercises, outdoor challenges and reflection sessions. Compared to the learning materials used in Sinha (in press), I therefore stripped presentation of math-related myths in the *growth mindset* intervention segment and added three new peer quotations from informal learning activities relevant to camping in the *utility value* intervention segment. Measurement-wise, I included new pre-post questionnaires on *learning goal orientation*, *attitude towards mistakes* and *utility of failure* to more directly capture dispositions, rather than solely relying on students’ open-ended responses as in Sinha (in press). I simplified explanations throughout the intervention materials and had teachers verify them for comprehensibility by lower secondary students, who may possess less developed reading skills than upper secondary students. Taken together, this conceptual replication maintained the original study’s theoretical hypothesis (*growth mindset* and *utility value* principles can improve student dispositions towards failure) but systematically altered a few components of the study design and administration to explore the generalizability and boundary conditions of the original findings across different developmental stages and intervention contexts.

### 2.5 Research questions

RQ1: How does students’ *learning goal orientation* change post-intervention?
RQ2: How does students’ *attitude towards mistakes* change post-intervention?
RQ3: How do students’ domain-general *beliefs about growth mindset* change post-intervention?
RQ4: How does students’ appraisal of the *utility of failure* change post-intervention?

## 3 Method

### 3.1 Participants

After obtaining informed consent from students and their parents, *N* = 68 lower secondary school students (grade 7, age 12–13) from a specialized independent school in Singapore participated. The sample comprised 68.1% males (*n* = 49), 19.4% females (*n* = 14), 11.1% (*n* = 8) not listed or preferred not to say, with 1.4% (*n* = 1) missing data for gender. Ethnicity-wise, the sample had 77.8% Chinese (*n* = 56), 8.3% Indian (*n* = 6), 12.5% (*n* = 9) not listed or preferred not to say, with 1.4% (*n* = 1) missing data for ethnicity. The majority of students (77.8%, *n* = 56) had scored 75% or more in their most recent standardized annual national examination for English. The study was approved by the ethics commission of Nanyang Technological University (IRB-2023-1040).

### 3.2 Procedure

**Figure 1:**
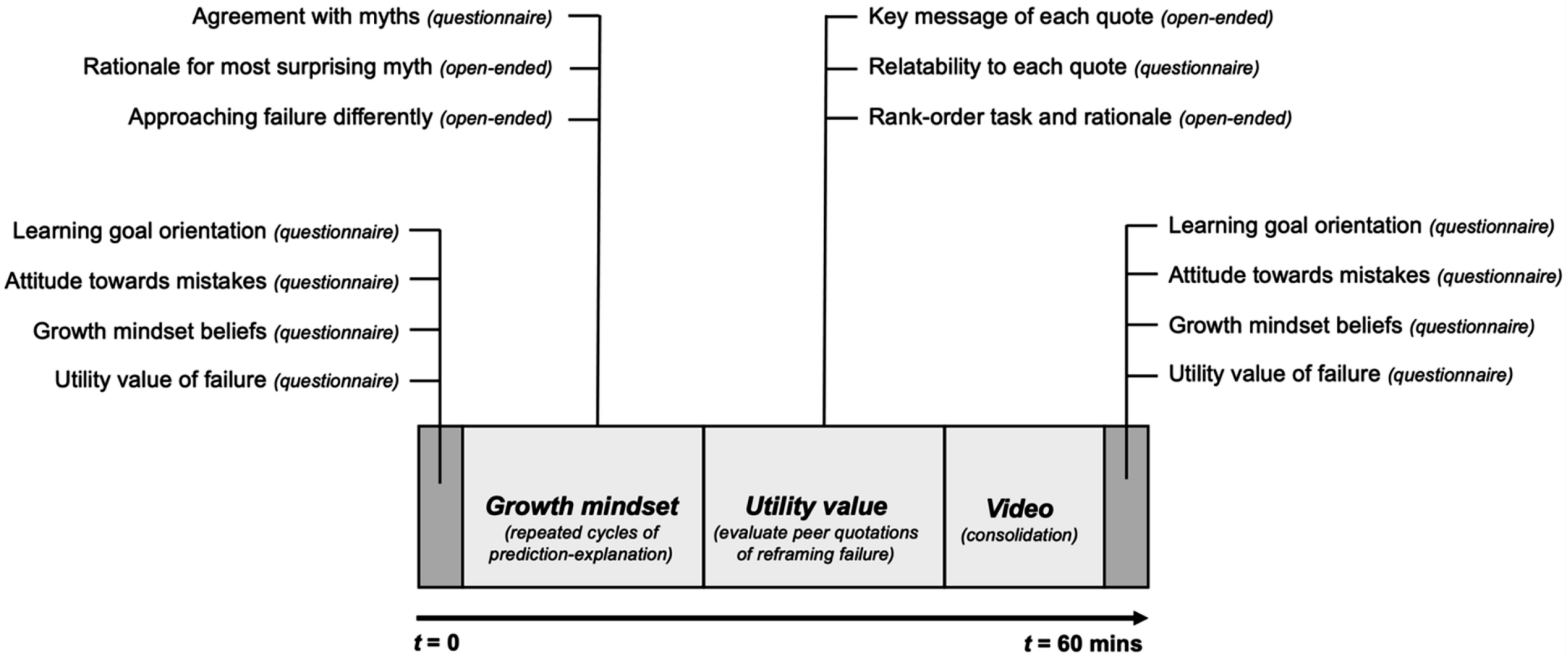
Study design.

I carried out a self-paced online study lasting up to 1 h (*M* = 30.6 min, *SD* = 9.3 min, *max* = 54.6 min) during classroom time. See Figure 1 for the study design.

All students started with exposure to a two-part interactive storyline on brain plasticity and the concept of growth mindsets, building upon and expanding the validated materials from Yeager et al. (2019). While Yeager et al. (2019) focused on having participants read scientific literature on neural plasticity, learn about the application of growth mindsets by students and celebrities, and engage in writing exercises to reinforce key concepts, the present online training materials were organized around common myths. Following Sinha (in press), in part I, students were therefore visually introduced to essential concepts involving neurons, synaptic connections. They made predictions regarding two brain myths associated with intelligence and failure (e.g., *“your brain cannot get smarter,” “when I fail, my brain grows bigger”*), receiving personalized feedback that emphasized the brain’s capacity for adaptability through practice that is driven by failure. Part II addressed the distinctions between domain-general growth mindsets and fixed mindsets—again via initial exposure to three myths (e.g., *“people with a growth mindset view failures as shortcomings (instead of opportunities for growth),” “a good growth mindset is just about effort—if you try hard enough, you are bound to improve,” “you only can have one type of mindset—either a fixed or growth mindset at a time”*)—encouraging students to anticipate viewpoints on failure, effort, and the types of mindsets while providing them with tailored follow-up feedback that highlighted the importance of persistence, effort, and improvement strategies. I also showed local academic achievement data from the 2018 PISA assessment via a bar graph along with a brief explanation demonstrating the downstream advantages of adopting a growth mindset (e.g., students from Singapore who believed their abilities could grow with effort and persistence scored, on average, 41 points higher in reading (8% improvement) compared to those who thought their abilities were fixed). This study segment wrapped up with reflective questions encouraging students to identify which growth mindset myth surprised them most and to elaborate how they would handle failure-driven learning experiences in the future.

In the next study segment, a variety of relatable peer quotations were shared to highlight the advantages of failure and to recontextualize its perceived costs within academic settings such as creative problem-solving and outdoor teamwork-focused activities, encouraging students to learn vicariously by reflecting on their own experiences. Following Sinha (in press), I utilized five quotations (three newly-designed) illustrating how failure (i) can enhance creativity, (ii) serve as a source of motivation, (iii) promote deeper engagement with content, (iv) provide chances to correct misconceptions, along with the (v) immediate and long-term benefits of experiencing failure. After engaging with these quotations, students ranked them from their most to least preferred and explained their top choice. Subsequently, a five-minute animated video was shown to introduce ideas of mental resilience for learning and reinforce key messages from each quote. Finally, students composed their own quotations directed at future peers. See Supplementary information for more details on the content of peer quotations used.

### 3.3 Measures

I collected pre-post measures of four key outcomes relevant to the study, all on a five-point Likert scale ranging from *strongly disagree* to *strongly agree*. First, *learning goal orientation*, via a validated 8-item questionnaire adapted from Button et al. (1996) comprising items such as *“the opportunity to do challenging work is important to me”* and *“I prefer to work on tasks that force me to learn new things.”* Second, *attitude towards mistakes*, via a validated 8-item questionnaire adapted from Leighton et al. (2015) capturing subdimensions of affect (e.g., *“when I fail to answer classroom questions, I am overwhelmed with embarrassment”*) and cognition (e.g., *“I believe successful students fail less during learning than others”*). Third, *beliefs about growth mindset*, via a validated 6-item questionnaire adapted from Zeeb and Voss (2025), with items such as *“mental ability is a part of a person that cannot really be changed”* and *“you can learn new things, but you cannot change basic mental performance.”* Fourth, *utility of failure*, via a 4-item questionnaire, again drawing on from Zeeb and Voss (2025), with items such as *“failures are useful for obtaining information about the quality of my learning”* and *“I find failures useful in connecting new information with what I already know.”* Based on the data sample, all four scales showed acceptable reliability pre-study (McDonald’s ω = 0.88, 0.81, 0.80, 0.75) and post-study (McDonald’s ω = 0.88, 0.83, 0.82, 0.61). Complete scales can be found in Supplementary information. The study concluded by capturing demographic data such as confidence in reading and writing skills (five-point Likert scale ranging from *strongly disagree* to *strongly agree*), scores from the most recent primary school leaving examination, gender, and ethnicity.

### 3.4 Analysis plan

Owing to the non-normal nature of the data (confirmed via a significant Shapiro–Wilk assumption check), a two-tailed paired samples Wilcoxon signed-rank test was used for evaluating pre-post changes in the four measures used. Rank-biserial correlation (r_rb_) was used as the non-parametric effect size measure. In cases of non-significant results, I used Bayes factor (BF_01_) to quantify strength of evidence favoring the null hypothesis, with BF_01_ between 3 and 10 typically suggesting positive/substantial odds (Jarosz and Wiley, 2014). Using BF_01_ allowed distinguishing cases where a non-significant result (*absence of evidence*) truly corresponded to *evidence of absence* from cases where it did not, thereby addressing a common criticism of statistical approaches that rely solely on null hypothesis significance testing (Cumming, 2014).

## 4 Results

### 4.1 Implementation fidelity

**Figure 2:**
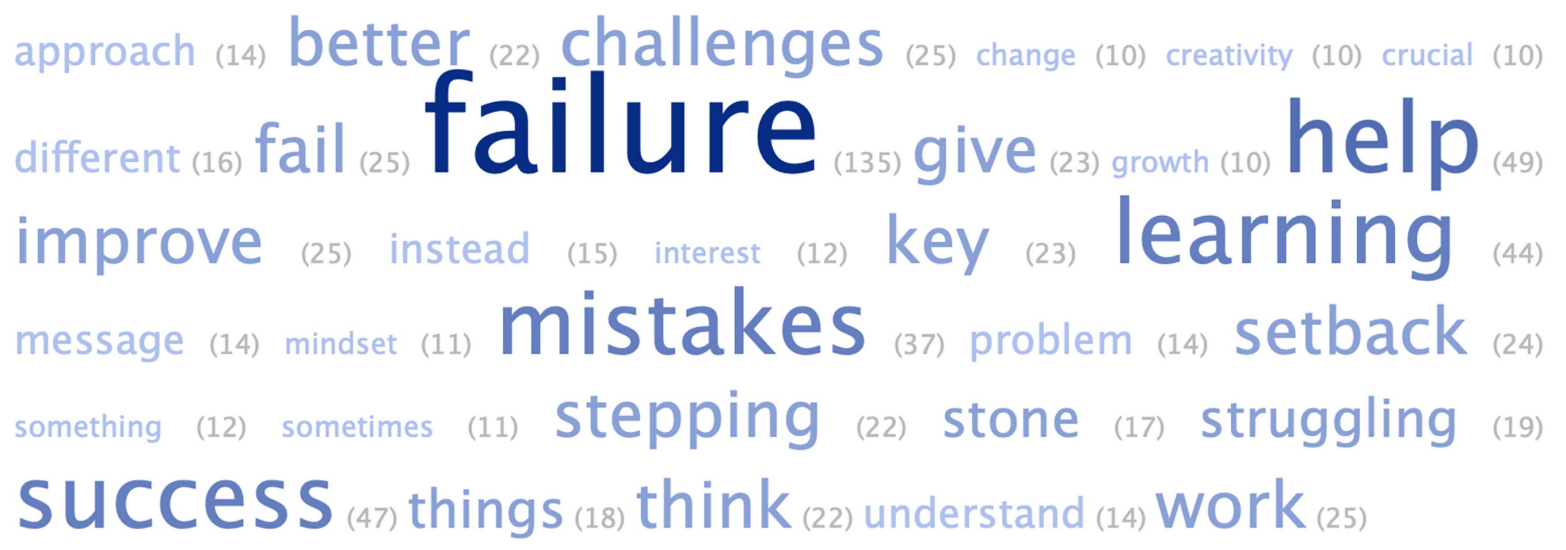
Word cloud from students’ elaboration of key messages they identified in peer quotes.

Students spent sufficient time across the two intervention segments, given they were designed to be brief and intuitive. The average duration for the *growth mindset* segment was 5.9 min (*SD* = 2.4 min, *max* = 12.1 min). For the *utility value* segment, students spent an average of 7.1 min (*SD* = 5.6 min, *max* = 40.8 min), engaging with each of the five quotes for an average of 1.2 min (*SD* = 1.7 min). Students were able to successfully articulate key messages from their reading of peer quotes, as seen from the word cloud distribution (along with frequencies) from Figure 2. In alignment with my expectations, post hoc deductive analysis (Fife and Gossner, 2024) showed that students’ peer quotation interpretations focused on verbalizing how failure (i) can enhance creativity (e.g., *“See failure as an opportunity to get better ideas,” “Failure can lead to new interests”*), (ii) serve as a source of motivation (e.g., *“Moments of failure motivate people to work harder and think outside the box and makes success sweeter,” “Accomplishment is not just about finishing, it’s about failure that motivates us to work harder”*), (iii) promote deeper engagement with content (e.g., *“Mistakes help deepen your understanding of something,” “Failure makes you think deeper and understand the task at hand”*), (iv) provide chances to correct misconceptions (e.g., *“Failure helps us figure out what would not work and improve by avoiding those mistakes”*), and the (v) immediate and long-term benefits of experiencing failure (e.g., *“Struggling does not mean that you are not improving, it means that you need a better approach”*). Across all *n* = 272 open-ended responses during both these intervention segments, students’ average word count was 31.65 words (*SD* = 25.08 words, *max* = 148 words), with all but *n* = 2 responses written in good faith (non-gibberish) reflecting an honest attempt to answer the reflection question. An invalid response would be any of these: “idk,” “I do not know,” “nothing,” “no,” “never,” or a response that stopped at the sentence starter (i.e., “dear struggling student,” “because my dad”) or a nonsense response (e.g., random letters and numbers). The rarity of such invalid responses signals high levels of cognitive engagement with the open-ended questions. On average, students spent *M* = 5.1 min viewing the five-minute consolidation *video* (*SD* = 2.9 min, *max* = 25.5 min).

### 4.2 Learning goal orientation (RQ1)

Students significantly improved their learning goal orientation after the intervention with a moderate effect—pre-intervention (*M* = 4.03, *SE* = 0.08) and post-intervention (*M* = 4.16, *SE* = 0.07, *z* = 2.08, *p* = 0.037, r_rb_ = 0.33). This effect (equivalent Cohen’s *d* = 0.70) corresponds to a moderate to strong effect based on Cohen’s benchmarks (Cohen, 1988), and is nearly 3.5x the estimate of having a very high-quality teacher (versus an average teacher) for one year (Hanushek, 2011). When translated into common language effect sizes, this effect reflects a 69% chance of a randomly picked student self-reporting a higher learning goal orientation post-intervention relative to pre-intervention.

### 4.3 Attitude towards mistakes (RQ2)

After undergoing the intervention, students also showed a significantly more positive attitude towards mistakes, with lower scores indicating better attitudes—pre-intervention (*M* = 3.14, *SE* = 0.09) and post-intervention (*M* = 2.87, *SE* = 0.09, *z* = −3.50, *p* < 0.001, r_rb_ = −0.52). This effect (equivalent Cohen’s *d* = 1.22) corresponds to a very strong effect based on Cohen’s benchmarks (Cohen, 1988), and reflects an 80.6% chance of a randomly picked student self-reporting an improved attitude towards mistakes post-intervention relative to pre-intervention.

### 4.4 Beliefs about growth mindset (RQ3)

In terms of beliefs about growth mindset, self-reported post-intervention scores (*M* = 3.69, *SE* = 0.11) were not significantly different from their pre-intervention scores (*M* = 3.59, *SE* = 0.11, *z* = 0.99, *p* = 0.325, r_rb_ = 0.15). Despite descriptively higher scores post-intervention, the sample showed strong odds favoring the null hypothesis of no belief change (BF_01_ = 5.09). This effect (equivalent Cohen’s *d* = 0.30) was adjudged as weak based on Cohen’s benchmarks (Cohen, 1988), and corresponded to only a 58.4% chance of a randomly picked student self-reporting more positive beliefs about growth mindset post-intervention relative to pre-intervention.

### 4.5 Utility of failure (RQ4)

Finally, I did not find a significant difference in students’ appraisal of the utility of failure pre-intervention (*M* = 3.78, *SE* = 0.09) and post-intervention (*M* = 3.93, *SE* = 0.08, *z* = 1.67, *p* = 0.094, r_rb_ = 0.27), however, only with very weak odds favoring the null hypothesis (BF_01_ = 1.46). This effect (equivalent Cohen’s *d* = 0.56) corresponds to a moderate effect based on Cohen’s benchmarks (Cohen, 1988), and reflects a 65.4% chance of a randomly picked student self-reporting a better appraisal of the utility of failure post-intervention relative to pre-intervention.

## 5 Discussion

I replicated a novel intervention integrating growth mindset and utility value principles to scaffold learning goal orientation, attitude towards mistakes, beliefs about mental ability and the utility of failure in lower secondary students. Everyone engaged in two primary learning activities—(i) making predictions on *growth mindset* myths followed by expository instruction on the canonical underlying ideas, and (ii) evaluating peer quotations of reframing failures across formal and informal learning contexts, which nudged reflection on the relevance and relatability of failures to own learning. Manipulation checks showed that the intervention served its goals.

Results for RQ1 and RQ2 showed that brief exposure to the intervention materials was sufficient to trigger significant improvements in students’ *learning goal orientation* and *attitude towards mistakes*. For instance, one student noted *“Last year, when I struggled with a tough math topic, I got frustrated and assumed I just wasn’t good at it. I avoided asking for help and moved on without fully understanding it. Now, I would approach failure as a chance to grow—I’d ask more questions, practice deliberately, and remind myself that struggling means I’m learning,”* while another student shared *“I remember a time during my primary IV camp where during the rock climbing activity, I kept on failing and falling down the rocks. In the end, I felt embarrassed and gave up as everyone was staring at me, waiting for their turn and I did not want to hold up the line. I would try my best and not give up if I were to do it again.”*

This is a noteworthy finding, given that such dispositional changes are often challenging to achieve and typically require sustained effort. Former empirical work (e.g., Geitz et al., 2015; Yokoyama and Miwa, 2021) has shown that changing goal orientation may not be straightforward with direct instruction, especially when taking into account the complexity of classroom learning—for instance, if task designs promote a mastery goal while evaluation practices emphasize performance goals, students may receive mixed messages (Benita and Matos, 2021; Liu et al., 2023). Ingrained beliefs about ability and the focus on competitive norms can further create resistance to adopting a learning goal orientation (Darnon et al., 2006). Students’ prior experiences and perceptions of classroom dynamics also play a significant role (Yu et al., 2025), making it challenging to shift their goal orientations without comprehensive and consistent changes across multiple classroom structures.

In her seminal work, Ames (1992) proposed how classroom structures may be modified to increase students’ orientation towards mastery, focusing on key domains like task design, evaluation, and authority (the TARGET framework). This involves (i) designing meaningful learning tasks that provide agency and opportunities for self-directed learning, (ii) creating evaluation practices that focus on individual progress and encourage intrinsically motivated efforts into learning tasks, and (iii) providing examples of mastery-oriented behaviors, such as taking on challenging tasks, persisting despite failure, and valuing learning for its own sake. The present work, which integrated growth mindset and utility value principles, was specifically designed to operationalize these core mastery principles with high fidelity through brief learning activities, thereby providing a theoretical explanation for the observed efficacy and rapid dispositional shifts. Specifically, I leveraged two primary mechanisms corresponding to Ames’s framework. First, by debunking the myth of fixed ability and providing students with a canonical understanding of growth mindset through prediction-making activities followed by expository instruction, the intervention offered students a new, mastery-oriented framework for interpreting ability, challenge, and effort (authority/recognition focus). Second, by using activities involving peer quotation evaluation that connected mistakes to data points for growth, the intervention provided not only an immediate replacement of the detrimental interpretation of failure with a mastery-oriented one but also offered crucial vicarious learning opportunities for students, making the adoption of similar attitudes relatable and accessible (evaluation/task focus). The resultant significant pre-post improvements with moderate effects support the premise that targeted, theoretically-grounded interventions can achieve rapid shifts in learning disposition when they provide a clear, actionable, and socially relevant cognitive framework for interpreting challenging experiences.

For instance, when students made predictions on myths prior to receiving instruction, they had the opportunity to be aware of gaps in their understanding of *growth mindset*, and their incorrect predictions may have induced surprise and motivated effort into reconciling that understanding—educational research into pedagogical designs based on desirable difficulties and productive failure (e.g., Bjork and Bjork, 2011; Kapur, 2014; Brod et al., 2018) aligns with this explanatory basis. The following two exemplar quotes provide further evidentiary support for this learning process—one student reflected on a nuanced understanding of effort beyond mere persistence, noting *“I originally thought that growth mindset is only about not being down after failure, by just putting more effort in the activity and that if you do so you would improve. However, I was proven wrong, you also need to try out more effective ways to improve if improvement is not shown.”* Similarly, another student provided a complementary perspective on the dynamic nature of mindsets by articulating *“I was really caught off guard by this myth because it makes it seem like we are trapped in one mindset forever, when in reality, we are constantly shifting. Some days, I feel unstoppable, like I can grow and improve endlessly. Other times, doubt creeps in, and I convince myself I’ll never be good enough. Realizing that mindsets aren’t set in stone is a relief—it means even in my lowest moments, there’s still a way forward.”*

On the other hand, observing behaviors and successful outcomes of similar others when navigating relatable learning situations of high failure likelihood may have reinforced students’ beliefs about mastery and mitigated the fear of failure. Foundational work on self-efficacy, especially regarding vicarious experiences (Bandura, 1977) explicitly discusses how observing the successes (and failures) of similar others in challenging situations can increase an observer’s self-efficacy beliefs and alleviate anxiety about failure, particularly when the modeled individual is perceived as relatable. Several students nonchalantly advised a reframed view of failure towards the end of the *utility value* intervention segment. For instance, one student articulated *“Do not worry about failing. Failing is natural, and it helps you learn what areas you have not done so well in, and even your strong suits….”* Another student echoed this sentiment emphasizing the instrumental role of setbacks (Narciss and Alemdag, 2025), noting that *“Failure is a process in which learning can occur smoothly. Without failure, you would not feel the need to be better, to work harder, to study smarter and fill in gaps in your learning….”* Such learning processes culminated in critical realizations such as *“Failure is not a bad thing, in life, we would always fail or make mistakes because we are still humans. It’s time to look at a things from a different perspective and have a growth mindset, it’s time to see the failures as opportunities to learn or stepping-stones to lead you to your final destination.”*

Results for RQ3 and RQ4, however, showed that the intervention materials did not significantly alter students’ beliefs about mental ability (intelligence) and the utility (value) of failure. Relative to upper secondary students for whom a significant improvement was obtained for belief change in Sinha (in press), this may suggest that younger student populations such as the current sample may need more explicit guidance and sustained reinforcement to transform their core beliefs. Owing to the exposure to a wider range of academic/social challenges and more developed reflective thinking, upper secondary students may be more receptive to belief change (King and Kitchener, 2004; Kuhn, 2009). An alternative explanation for these results stems from the sensitivity of the measurements—while items assessing *learning goal orientation* and *attitude towards mistakes* may have been more behaviorally focused and susceptible to immediate changes from the interventions’ practical strategies, examples and emotional reframing of failure, those assessing *beliefs about mental ability* and the *utility of failure* may have been relatively more abstract, necessitating deeper cognitive restructuring (Hong et al., 1999) and being less likely to be significantly affected within the short span of this intervention. Self-report scales assessing abstract motivational constructs have often been found to suffer from reduced reliability and social desirability bias (e.g., Credé and Phillips, 2011). A few other methodological explanations also warrant consideration for these non-significant results for RQ3 and RQ4—for instance, my construct operationalization may have targeted deep-seated cognitive schemas that necessitate longer or more immersive interventions for measurable change. Furthermore, the relatively brief duration of this intervention may have provided insufficient dosage for belief transformation. Prior work has shown that mindset and motivational interventions yield stronger outcomes when reinforced across multiple sessions or ecological contexts (Yeager and Dweck, 2012). Despite these non-significant results though, the observed descriptive trends were directionally consistent with theoretical expectations, creating space for future replications of this work, especially across more gender-balanced samples with varying ethnicities, as means to strengthen evidence for these potential underlying explanations.

## 6 Implications, conclusion and limitations

Taken together, these results implicate designing sensemaking-focused classroom activities that raise awareness of abstract ideas like growth mindset and provide concrete peer-based examples illustrating operational ways of rethinking about failure in learning. By integrating explicit (and anonymous) reflection opportunities, students may be well positioned to revise their goal orientations and attitude towards mistakes, as I could show from the empirical results. However, while this intervention may have planted the seeds for changes in beliefs about mental ability and the usefulness of failure, designing for sustained belief change would be an important future work avenue. What also remains to be seen yet is the downstream longitudinal impact of this psychological intervention on everyday classroom and school behaviors. Although I had deliberately designed the intervention to be brief so that it fits within packed secondary school curricula without significantly increasing teachers’ workload, the sustainability of the intervention would depend on offering redundant self-directed opportunities for students to scaffold their navigation with failure, especially prior to key academic milestones throughout the school term. Here, I chose a single-group pre-post design for pilot testing owing to the ethical constraints of withholding a potentially beneficial intervention, and for determining feasibility (can we deliver this novel intervention in secondary school settings?), acceptability (do students engage actively with the intervention materials?), and measurable changes (if any). However, I acknowledge that this design limits internal validity, and future replications of this validatory work are warranted in quasi-experimental or randomized designs for attributions of causality to individual intervention components (*growth mindset*, *utility value*). Measurement-wise, the data sample showed that the *utility of failure* scale’s reliability dropped from ω = 0.75 (pre-intervention) to ω = 0.61 (post-intervention). This differential reliability suggests the intervention may have altered the construct’s interpretation, thus potentially attenuating the observed effect size and highlighting a need for item refinement in future work.

## Data Availability

The raw data supporting the conclusions of this article will be made available by the authors, without undue reservation.
