# Supplementary material for: Improving cognition and perception towards failure: a conceptual replication study
Source: Front Psychol. 2025 Dec 8;16:1650136. doi: 10.3389/fpsyg.2025.1650136 (PMC12722885; doi:10.3389/fpsyg.2025.1650136)
Supplement: Supplementary file 1 [file Data_Sheet_1.pdf]

SUPPLEMENTARY MATERIALS

Complete scales used in the study

Learning goal orientation (5-point Likert scale from "strongly disagree" to "strongly agree")

1. The opportunity to do challenging work is important to me.
2. When I fail to complete a difficult task, I plan to try harder the next time I work on it.
3. I prefer to work on tasks that force me to learn new things.
4. The opportunity to learn new things is important to me.
5. I do my best when I'm working on a fairly difficult task.
6. I try hard to improve on my past performance.
7. The opportunity to extend the range of my abilities is important to me.
8. When I have difficulty solving a problem, I enjoy trying different approaches to see which one will work.

Attitude towards mistakes (5-point Likert scale from "strongly disagree" to "strongly agree")

1. When I fail, I am afraid that others look down upon me *[affect subscale]*
2. If I fail, I don't want others to notice them *[affect subscale]*
3. When I fail to answer classroom questions, I am overwhelmed with embarrassment *[affect subscale]*
4. I seldom feel bothered by my failures *[affect subscale] (reverse coded)*
5. I believe successful students fail less during learning than others *[cognition subscale]*
6. I believe it is smart to avoid failures/failing during learning *[cognition subscale]*
7. I believe failing is not an efficient way to learn academic materials *[cognition subscale]*
8. I believe failing may reduce my interest in learning *[cognition subscale]*

Beliefs about growth mindset (5-point Likert scale from "strongly disagree" to "strongly agree")

1. One has a certain level of mental ability that cannot be changed *(reverse coded)*.
2. Mental ability is a part of a person that cannot really be changed *(reverse coded)*.
3. You can even significantly change your basic mental abilities.
4. No matter who you are, you can significantly change your mental abilities.
5. You can learn new things, but you can't change basic mental performance *(reverse coded)*.
6. In essence, you can always change how intelligent you are.

Utility of failure (5-point Likert scale from "strongly disagree" to "strongly agree")

1. Failures are useful for obtaining information about the quality of my learning.
2. Learning from failures can help me approach new problems and plan self-study.
3. I find failures useful in connecting new information with what I already know.
4. I find it unnecessary for my future professional life to learn about how failures can be useful *(reverse coded)*.

SUPPLEMENTARY MATERIALS

Utility value intervention materials

	Peer Quotations	Detailed Rationale
1. Illustrates benefits of failure by showing that ***failure stimulates creativity***	*Before the egg drop challenge, science was a chore – memorizing facts with no real application. It wasn't engaging at all. But then, something amazing happened. Failing became the key to unlocking creativity. When designing contraptions to protect our eggs from breaking when dropped from a height, every broken egg wasn't a disappointment, but a stepping stone in a 'trial and error' process. We had to constantly think outside the box, coming up with new designs and material combinations to protect the egg. These repeated failures weren't setbacks, but opportunities to experiment and innovate. It was this constant push to find creative solutions that made the challenge so exciting, and the final success so rewarding. Science wasn't just about memorizing facts anymore; it was about using them creatively to solve problems. That's what truly sparked my interest in learning.*	**Highlights "trial and error" as creative problem-solving:** It emphasizes the "trial and error" approach as a way of fostering creativity.<br><br>**Reframes failure as an opportunity to experiment:** Instead of simply mentioning failure, it describes each broken egg as an opportunity to experiment with new ideas.<br><br>**Focuses on "outside the box" thinking:** It emphasizes how every failure pushed them to think outside the box and come up with new solutions.<br><br>**Connects failure to sparking interest:** It clearly connects the experience of failure to the student's newfound interest in learning science.
**2. Illustrates benefits of failure by showing ***failure as a motivator***	*The low beam challenge really pushed us as a group. We had to help each person cross without stepping off the beam, and at first, it seemed impossible. Each time someone lost their balance or stepped off, we had to pause and rethink our strategy. It was frustrating, but instead of giving up, each failure motivated us*	**"Dead end" reframed as a challenge:** Instead of simply mentioning failure, it describes hitting a dead end as a challenge they had to overcome.

	to find new ways to work together and problem-solve. We realized that failure wasn't a setback; it was a sign that we needed to adjust and try something different. Every misstep gave us a better understanding of what wouldn't work, which brought us closer to figuring out what would. By the time, we made it across, the sense of accomplishment wasn't just about finishing – those moments of failure actually motivated us to work harder and think outside the box, making the success even sweeter.	**Emphasis on increased determination:** It emphasizes how facing failure fueled their determination to find new solutions.<br><br>**Success after overcoming challenges:** The success is framed as more rewarding because it came after overcoming challenges, highlighting the motivational aspect of failure.
3. Reframes the cost of failure by revealing its ***benefits of deeper engagement***	*Exams used to be anxiety bombs – the pressure to get the right answer froze my brain. I'd get frustrated and lost, hating the feeling of not knowing. But then, I noticed something interesting: the mistakes I made actually fueled my curiosity. Every wrong answer sparked a deeper interest in understanding the concept behind it. Fixing those misunderstandings wasn't just about getting the right answer, it was about truly grasping the 'why.' And guess what? The more engaged I became with figuring things out, the better I retained the information. Now, I don't see mistakes as failures, but as stepping stones to deeper learning. Strangely enough, I almost welcome them now during my studies, because I know they'll ultimately lead to a stronger understanding and better exam performance.*	**Mistakes as sparks for curiosity:** Getting things wrong became a trigger for deeper exploration and understanding.<br><br>**Focus on "why" not just "what":** Fixing mistakes wasn't just about getting the correct answer, but about truly understanding the underlying concepts.<br><br>**Deeper engagement leads to better retention:** The act of figuring things out through mistakes led to a stronger grasp of the material.<br><br>**"Welcome" mistakes for deeper learning:** The student now sees mistakes as an opportunity for deeper

		engagement, not something to be avoided.
**4. Reframes the cost of failure by demonstrating ***strategic failing as opportunities for rectifying misinformation***	*When we faced the giant wall at camp, it seemed impossible at first. My first few attempts were total failures – I couldn't even get halfway up. But instead of seeing it as defeat, I realized those failures helped me figure out what wouldn't work. Each time I fell short, it taught me what to avoid and how to adjust my approach. I learned where to place my hands, how to position my feet, and how to better coordinate with the group. Every failure became a clue, guiding me towards a better strategy. Eventually, after a few tries, we all made it over the wall. It wasn't just about crossing it – it was about understanding the process and learning what works and what doesn't. In the end, failure didn't just show us where we were going wrong, it helped us recognize the path forward. That experience taught me that failure is not just a setback – it's a crucial part of discovering what works and finding success.*	**Misinformation exposed:** Mistakes are seen as red flags highlighting areas where the student has misconceptions.<br><br>**"Detective Story" analogy:** The process of identifying and correcting the root cause of the mistake is framed as a detective story, making it more engaging.<br><br>**Focus on learning, not number of mistakes:** The emphasis shifts from the negativity of making mistakes to the positive outcome of gaining a deeper understanding
**5. Reframes the cost of failure by showcasing its ***immediate process benefits*** and ***delayed outcome benefits***	*Moving from Primary 6 to Secondary 1 was overwhelming – suddenly, there were so many subjects, and everything moved so much faster. I wasn't just struggling with a few mistakes; I felt like I couldn't keep up at all. No matter how hard I tried, there were always more concepts to grasp, more assignments piling up, and I wasn't performing the way I used to. It was frustrating. It felt like failing, and I started to doubt if I could ever catch up. But then, I realized that struggling didn't mean I wasn't*	**Wrong answers revealed knowledge gaps:** Each mistake became a learning opportunity, highlighting areas where they needed to focus.<br><br>**Process benefits:** The act of tackling past papers itself fostered critical thinking and deeper understanding, making learning more engaging.

	improving – it meant I needed a better approach. Instead of just pushing through the same way, I started identifying which topics I was weakest at and focusing on those first. I changed how I took notes, practiced active recall, and asked for help when needed. Slowly, I stopped feeling like I was falling behind and started seeing my struggles as checkpoints, not roadblocks. Looking back, those challenges helped me get better at managing heavier workloads.	**Delayed outcome benefits:** The initial struggles led to a more solid grasp of the material in the long run.

Note: Quotes 2, 4 and 5 were newly-designed keeping lower secondary schooling contexts in mind.

SUPPLEMENTARY MATERIALS

Consolidation video

Link to the video: https://www.youtube.com/watch?v=hD0rtFo6YKI

Follow-up attention check questions:

Question 1: What does Kevin do before the lecture that is an example of an effortful learning strategy?

a) He attempts the tutorial questions
b) He watches a documentary on a topic
c) He asks a friend for help
d) He reads the textbook chapter

Correct Answer: a)

Question 2: What do the voices of doubt try to convince Kevin of?

a) That he is not smart enough to succeed
b) That studying would be easy
c) That he should ask for more help
d) That he should give up on his dreams

Correct Answer: b)

Question 3: What does Kevin learn about effortful learning as he climbs Challenge Peak?

a) It is always the easiest way to learn
b) It should be done alone without distractions
c) It is only effective for certain subjects
d) It is okay to ask for help sometimes

Correct Answer: d)

Question 4: What example is given of how Tyler engaged in effortful learning?

a) He spent hours studying every night
b) He learned from repeated failures in the egg drop challenge
c) He always asked for help when he was struggling
d) He took detailed notes during every lesson

Correct Answer: b)

Question 5: What does the video say is the ultimate reward of effortful learning?

a) Getting food grades
b) Impressing teachers
c) A deeper understanding that endures
d) Avoiding failure

Correct Answer: c)
